# Significance of *Clostridium difficile* in community-acquired diarrhea in a tertiary care center in Lebanon

**DOI:** 10.1038/s41598-020-62418-9

**Published:** 2020-03-30

**Authors:** Reem Al Assaad, Alik Dakessian, Rana Bachir, Abdul Rahman Bizri, Mazen El Sayed

**Affiliations:** 1 Department of Emergency Medicine, American University of Beirut Medical Center, Beirut, Lebanon; 2 Department of Internal Medicine, Division of Infectious Diseases, American University of Beirut Medical Center, Beirut, Lebanon; 3 Emergency Medical Services and Pre-hospital Care Program, American University of Beirut Medical Center, Beirut, Lebanon

**Keywords:** Gastroenteritis, Clostridium difficile

## Abstract

*Clostridium difficile* infection (CDI) is becoming a cause of community-acquired diarrhea. The aim is to describe (CDI) as a cause of acute diarrhea in patients presenting from the community to the Emergency Department (ED) of a tertiary care center in Lebanon. A retrospective study conducted in the ED at the American University of Beirut Medical Center (AUBMC). Adult patients presenting with the chief complaint of diarrhea and having positive CDI by stool laboratory testing (toxins A and B), during a three-year period were included. 125 patients with CDI were included. Average age was 61.43 (±20.42) with roughly equal sex prevalence. 30% (n = 36) of patients had neither antibiotic exposure nor recent hospitalization prior to current CDI. Mortality was 9.6% (n = 12), CDI was attributed as the cause in 16.7% (n = 2) and a contributing factor in 41.6% (n = 5). Recurrence within 3 months occurred in 9.6% (n = 11) in mainly those taking Proton Pump Inhibitors (PPIs) and having multiple co-morbidities. There is a high rate of community acquired CDI in Lebanon. Review of patients’ medications (PPIs and antibiotics) is crucial. More studies are needed to assess mortality associated with CDI and the outcome of coinfection with other enteric pathogens.

## Introduction

*Clostridium difficile* (*C. difficile*) is a spore forming, Gram positive, anaerobic bacterium that produces two main toxins: enterotoxin A and cytotoxin B, both of which have been incriminated in the pathophysiology of diarrhea caused by this bacterium^1^. It is the leading cause of hospital-acquired diarrhea, with prolonged hospitalization, age above 65 years, antibiotic usage, underlying medical conditions, neoplastic disease, gastrointestinal surgery, nasogastric tubes, and gastrointestinal disorders being well established risk factors associated with the development of nosocomial *C. difficile* infection (CDI)^2^.

While the role of *C. difficile* in diarrheal disease in hospitalized patients has been well established, there is little information on its role as a cause of community acquired diarrhea. Recent reports suggest that the occurrence and severity of *C. difficile* associated disease in the community and in children is increasing^3^. The financial burden of this illness is significant because of the need for hospitalization and possibility of recurrence^4^. The spectrum of CDI ranges from mild illness to severe fulminant inflammatory colitis^5^. Three possible causes for community acquired *C. difficile* diarrhea have been outlined including colonization of the gastrointestinal tract of patients recently discharged from hospital, increased spread of *C. difficile* within hospitals leading to an increased rate of asymptomatic carriage in the population; and contact with asymptomatic carriers^5^.

The epidemiology of CDI is evolving. The organism is increasingly becoming a cause of community acquired diarrheal illness in persons who are young and otherwise healthy^3^. Thus, more research is needed to determine the epidemiology, risk factors, control measures, and effective treatment strategies of this emerging entity.

The aim of our study is to describe *C. difficile* infections among patients presenting with diarrhea to the Emergency Department (ED) of a tertiary care center in Beirut Lebanon, and to identify patients’ characteristics and clinical findings associated with this illness.

## Materials and Methods

This retrospective study was conducted in the ED at the American University of Beirut Medical Center (AUBMC). AUBMC is the largest academic tertiary care medical center in Lebanon, and a major referral center for Lebanon and the region.

**Figure 1:**
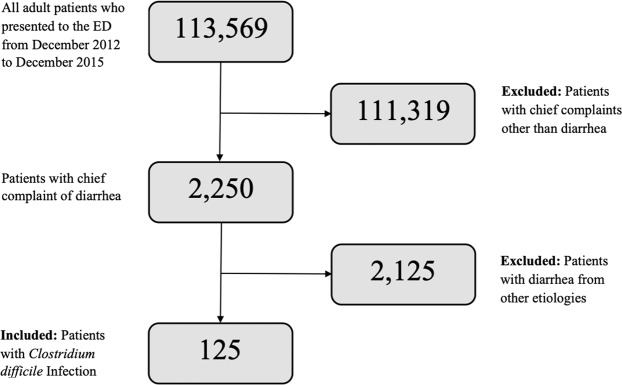
Inclusion and Exclusion Flowchart.

A chart review was done on adult patients ≥18 years of age who presented to the ED with the chief complaint of diarrhea and had a positive CDI during a three-year period (December 1, 2012 to December 1, 2015) (Fig. 1). The diagnosis of CDI was in accordance with the Clinical Practice Guidelines set forth by Infectious Diseases Society of America (IDSA), whereby stool laboratory testing for toxins A and B was done as a confirmatory test after a positive Glutamate Dehydrogenase (GDH)^6^. Variables collected included: demographics, history of exposure to individuals with CDI, prior history of CDI, antibiotic use within the past 3 months, type of antibiotic (if applicable), prior history of hospitalization and surgeries, other contributing factors (co-morbidities, intake of proton pump inhibitors (PPIs), laboratory studies and imaging, disposition and recurrence within 3 months).

The Institutional Review Board (IRB) of the American University of Beirut approved this study and all methods were carried out in accordance with relevant guidelines and regulations. Data was collected by trained research fellows from the ED and inpatient medical records department. A waiver for the need for informed consent was obtained as part of the IRB approval for this retrospective study.

To maintain the inter-rater reliability of the retrospectively collected data, a coding manual was created defining the study variables and their location in the patients’ charts. Moreover, data collection was completed by two trained post medical doctoral research fellows. Two strategies were implemented to ascertain the inter-rater reliability. First, a pilot study was conducted on 10% of the sample to assess the degree of reliability of the data collection sheet and to determine the availability of the variables and to adopt the best method for data abstraction. Then, the principal investigator regularly reviewed a random sample of the completed records and assessed to what extent the collected information is accurate and solved any identified data conflict through discussion with the research fellows until reaching consensus.

A descriptive analysis was carried out for the study sample. All categorical variables were summarized by calculating their frequencies and percentages. Age was presented as mean ± standard deviations and range. Statistical analyses were performed using SPSS 24 (Statistical Package for Social Sciences).

## Results

A total of 125 cases of CDI out of 2250 patients presenting with diarrhea were identified yielding a rate of 5.5%. The mean age was 61.45 years (±20.42) and 52% of the patients were of female sex. More than half of the patients (n = 74, 59.2%) were hospitalized within the last 3 months with most (n = 66, 89.2%) discharged less than 1 month before the current CDI. Over 40% (n = 51) of patients were not hospitalized and 44% (n = 55) did not have antibiotic exposure (excluding Vancomycin and Metronidazole) in the past 3 months. In patients with antibiotic exposure, carbapenems were the most common agents (25.7%) followed by fluoroquinolones (24.3%). Almost 30% of patients were neither hospitalized nor were they taking antibiotics in the past 3 months prior to the current CDI. The majority of patients (92.8%) did not undergo any surgical procedures in the 1 month prior to CDI. (Table 1).

**Table 1:** Demographics & characteristics of patients with CDI.

Age	Mean ± SD	Range
N = 125	61.43 ± 20.42	19–94
	Frequency	Percentage
**Sex**		
Male	60	48.00%
Female	65	52.00%
**Hospitalized within the past 3 months**		
Yes	74	59.20%
No	51	40.80%
**Discharge timeline of last admission**		
<1 month before current CDI	66	89.20%
1 to 3 months before current CDI	8	10.80%
**Antibiotic intake (excluding Vancomycin and Metronidazole) within the past 3 months**		
Yes, <1 month before current CDI	58	46.40%
Yes, 1–3 months before current CDI	12	9.60%
No	55	44%
**Type of Antibiotic**		
Carbapenems	18	25.70%
Fluoroquinolones	17	24.30%
Third Generation Cephalosporins	13	18.60%
Piperacillin-tazobactam	11	15.70%
Other	31	44.30%
**Neither hospitalized nor took antibiotics in the last 3 months**		
Yes	36	28.80%
No	89	71.20%
**Underwent a surgical procedure within the past 1 month**		
Yes	9	7.20%
No	116	92.80%

Stool cultures for bacteria other than *C. difficile* were done for almost all patients (93.6%), and only 4 patients had positive cultures, two of which were *Shigella sonnei*, one *Salmonella enteritidis* and one other non-typable species of *Salmonella*. Sigmoidoscopy was performed in only 6 patients without detecting pseudomembranes or ulcerations, whereas CT scan of the abdomen was performed in 28.8% of patients with active CDI. Colonic wall thickening was the most common detected abnormality on CT (55.6%), followed by peri-colonic fat stranding (33.3%) and bowel distension in 11.1%. In one third of the patients (33.3%) who underwent CT scanning during an active episode of CDI, no abnormal findings were found. (Table 2).

**Table 2:** Further tests & imaging.

	Frequency	Percentage
**Stool culture done**		
Yes	117	93.60%
No	8	6.40%
**Stool culture growth**		
No growth	113	96.60%
*Shigella sonnei*	2	1.60%
*Salmonella enteritidis*	1	0.80%
*Salmonella* spp nontypable	1	0.80%
**Was Sigmoidoscopy/colonoscopy performed during CDI?**		
Yes	6	4.80%
No	119	95.20%
**Pseudomembranes on sigmoidoscopy/colonoscopy?**		
No	6	100%
**Ulcerations on sigmoidoscopy/colonoscopy?**		
No	6	100%
**Was a CT scan of abdomen done during CDI?**		
Yes	36	28.80%
No	89	71.20%
**Results of CT scan**		
Colonic wall thickening	20	55.60%
Peri-colonic fat stranding	12	33.30%
Bowel distension	4	11.10%
No abnormal findings	12	33.30%

As far as disposition is concerned, 2 patients (1.6%) died at the ED, 11 (8.8%) were admitted to intensive care unit (ICU) and 99 (79.2%) to regular floor. Total number of patients who died were 12 (9.6%) with CDI was attributed as the cause in 16.7% and contributing factor in 41.6%. (Table 3).

**Table 3:** Deaths & dispositions.

	Frequency	Percentage
**Disposition**		
Home	12	9.60%
Home against medical advice	1	0.80%
Admitted to ICU	11	8.80%
• Death in ICU	2	
Admitted to regular floor	99	79.20%
• Death on regular floor	8	
Death in ED	2	1.60%
**Deaths Caused by CDI**	2	16.70%
**Deaths Contributed by CDI**	5	41.60%
**Deaths not related to CDI**	5	41.60%

Recurrence of CDI within 3 months occurred in 9.6%. The majority of those patients (72.7%) were taking Proton Pump Inhibitors and had co-morbidities like hypertension (54.5%) and diabetes mellitus type II (54.5%). Over half of those patients had 5 or more co-morbid conditions. Antibiotic use was found in 27.3% of patients who had recurrence. (Table 4).

**Table 4:** CDI recurrence & contributing factors.

	Frequency	Percentage
**Recurrence within 3 months following the onset of the previous episode**		
Yes	11	9.60%
No	104	90.40%
**Contributing Factors**		
PPI (Proton Pump Inhibitor)	8	72.70%
Hypertension	6	54.50%
Diabetes Mellitus Type 2	6	54.50%
Antibiotics (excluding Vancomycin and Metronidazole)	3	27.30%
Percentage of patients having 5 or more co-morbidities	6	54.50%

*1 patient recurred three times.

Other co-morbidities: history of CVA (Cerebrovascular Accident), CAD (Coronary Artery Diease), DL (Dyslipidemia), Atrial Fibrillation, Liver Cirrhosis, Heart Failure, DVT (Deep Venous Thrombosis), Ischemic Gastric Ulcer, CKD (Chronic Kidney Diease), Anemia.

## Discussion

Although many studies have aimed at describing the demographics, characteristics and outcomes of patients infected with *C. difficile* in the developed world, this is the first one that addresses this topic in Lebanon. Moukhaiber *et al*. and Berger *et al*. focused on the toxin types and molecular characterization of isolates from the same tertiary care center as our study^7,8^. Pechal *et al*. addressed the trends of CDI among each age group in United States community hospitals and found that the incidence was highest among individuals aged 74-85^9^, which is quite different from the mean age of 61.45 in our study. On the other hand, a study conducted in Kenya by Oyaro *et al*. concluded that there was a high incidence of CDI among younger adults, their average being 35.5 years with a range from 3–86^10^. It is important to mention that our study was based on adults and thus any patients who were under 18 years of age were excluded, which provides an explanation as to why the mean age between both studies is so different. Nonetheless, 91% of patients who tested positive in the study conducted in Kenya were under the age of 60 and infection with Human Immunodeficiency Virus (HIV) was a contributing factor in the prevalence of high CDI in that population^10^. HIV was not encountered in any of the patients in our study. Interestingly, Shin *et al*. found that younger individuals with less co-morbidities and no previous antibiotic use were more likely to acquire CDI from the community^11^.

In our study, approximately 30% of patients were neither hospitalized nor were they taking antibiotics in the past 3 months. These findings are not in line with previously established risk factors for acquiring CDI^12^. Since the definition community acquired CDI is when the onset of symptoms occurs within 48 hours of admission to the hospital or 1-month post discharge^13^, almost one third of the study patients acquired this infection from the community. Kutty *et al*. concluded that CDI was community acquired in 20% of cases in 2010^14^, which is lower compared to our study. In a meta-analysis made by Brown *et al*., it was concluded that patients who were taking Fluoroquinolones or CMCs (Cephalosporins, Monobactams and Carbapenems) were 5.5 and 5.68 times more likely to develop CDI respectively^15^. In our study Carbapenems and Fluoroquinolones were the 2 most commonly used antibiotics in the past 3 months prior to CDI. They concluded that patients taking Clindamycin had the highest risk to developed CDI^15^, but none of the 125 patients in our study had been using that antibiotic.

There are few studies regarding co-infection of *C. difficile* with other enteric bacteria like *Salmonella* and *Shigella*. It was somewhat described by Liao *et al*. where the age of the patients who were infected were no more than 2 years old^16^, whereas the 4 patients who had positive stool cultures in our study were adults. With regards to findings on CT scans, Kirkpatrick and Greenberg found that colonic wall thickening was seen in 76% of patients with active CDI, and pericolonic fat stranding in 57%^17^ compared to 55.6% and 33.3% respectively in our study. The differences in these results could be due to the much smaller sample size, where only 36 of patients in our study underwent CT scanning during active CDI, as opposed to 54 patients in their study. In another study by Boland *et al*., 39% of patients with CDI had no abnormal findings on CT^18^, comparable to our 33.3%.

Regarding mortality attributed to *C. difficile*, Hota *et al*. conducted a study and found that in patients with active CDI, it was the primary cause of death in 6% and a primary/secondary cause in 37%^19^. In our study, those percentages were 16.7% and 41.6% respectively. Differences could be explained by vastly different sample sizes or possibly late presentations to the hospital after onset of illness.

A systematic review found that the median overall risk of CDI recurrence was 22%^20^, which was greater than our rate of 9.6%. Moreover, Deshpande *et al*. reported the significance of the use of PPIs in recurrence^21^ and in our study 72.7% of patients who had CDI recurrence were on PPIs. Diabetes, which is a known risk factor for CDI recurrence^22^, was present in 54.5% of the study patients. Reveles *et al*. also found that hypertension was the most common co-morbidity found in those who had CDI recurrence^23^. Although hypertension was prevalent in 54.5% of patients who recurred in our study, the significance of such association is in question, due to the high prevalence of hypertension, reaching 36.9% in Lebanon^24^ and 31.1% in the world^25^. The use of antibiotics before admission was found to have statistically significant association in patients who had recurrent CDI after adjusting for confounders^26^, which was also evident in our study where almost 30% of patients who recurred were taking antibiotics prior to current infection. The significance of these co-morbid conditions in patients who had recurrence CDI in our study is unclear due to their small numbers.

This study was conducted by retrospective chart review. Testing for concomitant enteric viral or parasitic infections were not performed. Poor documentation is a major drawback with regards to data quality. Other exacerbating factors include improper charting in addition to illegible handwriting. Moreover, the data collected in order to conduct this study was from one tertiary care center in Lebanon. Other hospitals in Lebanon might report different experience with community acquired CDI. Collecting data related to emerging infections from all hospitals in Lebanon should be encouraged by the health authorities to detect potential epidemics.

Even with the aforementioned drawbacks, this study is the first that describes the demographics, characteristics and outcomes of patients with community acquired CDI in Lebanon, and one of very few in the region. This will prove to be a stepping stone for further studies to be conducted on the topic.

There is a high rate of community acquired CDI. Care should be taken in keeping CDI in the differential diagnoses of patients presenting with diarrhea, even in the absence of any known risk factors.

Since there was a lack of data regarding co-infection of *C. difficile* with other enteric bacteria in the adult population, more studies should be done in order to assess the severity of CDI in those patients, with potential differences regarding treatments and outcomes.

The importance of reviewing patients’ medications cannot be overemphasized. Those who are taking antibiotics should be followed up. Moreover, physicians need to be wary of the risk of using of PPIs in patients recently diagnosed with CDI.

It is evident in our study that CDI not only carries significant mortality but is also a contributing factor to death from other causes. However, the total number patients who did not survive were too small to draw any definite conclusions. There is room for further research on the mortality of CDI in the region.

### Ethical approval

The Institutional Review Board (IRB) of the American University of Beirut approved this study.

### Informed consent

Part of the exemption provided for this retrospective study by the IRB at the American University of Beirut, a waiver for an informed consent for review and analysis of existing data was obtained.
